# Reduced Metabotropic Glutamate Receptor Type 5 Availability in the Epileptogenic Hippocampus: An *in vitro* Study

**DOI:** 10.3389/fneur.2022.888479

**Published:** 2022-07-22

**Authors:** Maria Zimmermann, Luciano Minuzzi, Arturo Aliaga Aliaga, Marie-Christine Guiot, Jeffery A. Hall, Jean-Paul Soucy, Gassan Massarweh, Salah El Mestikawy, Pedro Rosa-Neto, Eliane Kobayashi

**Affiliations:** ^1^Department of Neurology and Neurosurgery, Montréal Neurological Institute, McGill University, Montréal, QC, Canada; ^2^Translational Neuroimaging Laboratory, Douglas Research Institute, McGill University, Montréal, QC, Canada; ^3^Department of Psychiatry and Behavioural Neurosciences, McMaster University, Hamilton, ON, Canada; ^4^PET Unit, McConnell Brain Imaging Centre, Montréal, QC, Canada; ^5^Department of Pathology, Montréal Neurological Institute, Montréal, QC, Canada; ^6^Department of Psychiatry, Douglas Research Institute, McGill University, Montréal, QC, Canada

**Keywords:** temporal lobe epilepsy, mGluR5, hippocampus, [^3^H]ABP688, autoradiography

## Abstract

Abnormalities in the expression of metabotropic glutamate receptor type 5 (mGluR5) have been observed in the hippocampus of patients with drug-resistant mesial Temporal Lobe Epilepsy (mTLE). *Ex-vivo* studies in mTLE hippocampal surgical specimens have shown increased mGluR5 immunoreactivity, while *in vivo* whole brain imaging using positron emission tomography (PET) demonstrated reduced hippocampal mGluR5 availability. To further understand mGluR5 abnormalities in mTLE, we performed a saturation autoradiography study with [^3^H]ABP688 (a negative mGluR5 allosteric modulator). We aimed to evaluate receptor density (B_max_) and dissociation constants (K_D_) in hippocampal mTLE surgical specimens and in non-epilepsy hippocampi from necropsy controls. mTLE specimens showed a 43.4% reduction in receptor density compared to control hippocampi, which was independent of age, sex and K_D_ (multiple linear regression analysis). There was no significant difference in K_D_ between the groups, which suggests that the decreased mGluR5 availability found *in vivo* with PET cannot be attributed to reduced affinity between ligand and binding site. The present study supports that changes within the epileptogenic tissue include mGluR5 internalization or conformational changes that reduce [^3^H]ABP688 binding, as previously suggested in mTLE patients studied *in vivo*.

## Introduction

Epilepsy is a neurological disorder characterized by recurrent, unprovoked seizures resulting from an imbalance between excitatory and inhibitory neural systems that afflicts ~1% of the general population (1, 2). Temporal lobe epilepsy (TLE) is characterized by an epileptogenic focus within the temporal lobe of the brain, most commonly within the mesial temporal structures, and more frequently so within the hippocampus (3). Seizures originating from the mesial temporal structures in mesial TLE (mTLE) present a high rate of intractability, for which suitable patients can be offered surgical treatment (4, 5).

Metabotropic glutamate receptor type 5 (mGluR5) is a G-protein coupled receptor expressed as a dimer primarily in the periphery of the post-synaptic terminal (6, 7). Each protomer includes a large extracellular Venus flytrap domain that contains the orthosteric binding site for glutamate. When glutamate binds to the receptor, a conformational change is induced that activates the G protein, initiating multiple signaling cascades, including several involved in gene regulation, enzymatic function, and release of intracellular Ca^2+^ stores (6, 7). mGluR5 has been shown to mediate neuronal excitability and promote synaptic plasticity under normal physiological conditions (8). In particular, the receptor is involved in the establishment of long-term potentiation and depression (9, 10).

Various lines of evidence have pointed to mGluR5 as a protein of interest in the study of epileptogenesis. The receptor has been implicated in the generation of ictal events and persistent neuronal hyperexcitability (11, 12). Furthermore, abnormal expression of mGluR5 has been identified in rat models of the disorder and in mTLE patients, through *in vivo* and *ex vivo* studies (13–19). Immunohistochemistry (IHC) analyses in mTLE surgical specimens have demonstrated mGluR5 upregulation in the hippocampus, independent of a neuropathological diagnosis of hippocampal sclerosis (13–15). In the pilocarpine rat model of mTLE, IHC and immunofluorescence data revealed decreased expression of hippocampal mGluR5 following status epilepticus, which was associated with a loss of mGluR5-dependent long-term depression (16, 17). In an amygdala-kindling rat model of TLE, reduced mGluR5 mRNA expression was detected in the hippocampus 24 h after the last kindled seizure (20).

The development of the positron emission tomography (PET) tracer [^11^C]ABP688 expanded the investigation of the role of this neuroreceptor in epileptogenesis by providing a non-invasive means to evaluate whole brain mGluR5 expression *in vivo*. [^11^C]ABP688 is a highly-selective and high-affinity non-competitive antagonist of mGluR5 that binds to its transmembrane allosteric site, comprising TMIII and TMVII (21–25). [^11^C]ABP688 PET studies have demonstrated reduced [^11^C]ABP688 binding potential (BP) within the epileptogenic hippocampi of mTLE patients, and within the hippocampus and amygdala of a pilocarpine-induced mTLE rat model (18, 19). Similarly, reduced [^11^C]ABP688 BP was found within the borders of focal cortical dysplasia, another intrinsically epileptogenic lesion frequently diagnosed in epilepsy patients (26).

Reconciling hippocampal mGluR5 *in vivo* [^11^C]ABP688 PET and *ex vivo* IHC data to date remains challenging, as these techniques significantly differ in their methods of receptor quantification, each providing a snapshot with very different spatial resolution. Whereas reduced *in vivo* [^11^C]ABP688 BP might at first seem in disagreement with *ex vivo* reports of increased immunoreactivity within the same brain regions, the dynamic conformational changes taking place *in vivo* must be recalled. It has been speculated that *in vivo* decreases in [^11^C]ABP688 BP in mTLE hippocampi may represent downregulation or internalization of mGluR5, unavailability of the [^11^C]ABP688 allosteric binding site due to conformational changes induced by persistent tissue hyperexcitability, or excess glutamate-induced changes to its binding affinity that could take place despite an upregulation and overexpression of the receptor (18).

To further shed light on the results of [^11^C]ABP688 PET studies, as well as to further support a role of mGluR5 in epileptogenesis, we conducted saturation autoradiography with [^3^H]ABP688 in surgically resected hippocampi of drug-resistant mTLE patients. We compared mTLE hippocampi with non-epileptic post-mortem hippocampal specimens from necropsy, to evaluate changes in mGluR5 density and [^3^H]ABP688-mGluR5 binding affinity *ex vivo*.

## Materials and Methods

This study was approved by the institutional REB.

### Samples Preparation

Hippocampal specimens were obtained from 16 drug-resistant mTLE patients (aged 26–67 at time of surgery; 11 female) who underwent surgical resection of their epileptogenic hippocampi at the Montréal Neurological Hospital after standard of care pre-surgical evaluation at the Epilepsy Monitoring Unit (Table 1). All patients provided written informed consent for research involving their removed brain tissue. Flash-frozen specimens were obtained from the Neuropathology Department.

**Table 1:** Patients clinical characteristics.

**ID**	**Gender**	**Epilepsy onset (years)**	**MRI diagnosis**	**EEG interictal/** **ictal**	**Age at surgery**	**Surgery**	**Duration of epilepsy (years)**	**ASM at surgery**	**Pathology**	**Engel class**	**Follow-up: years from (last) surgery**	**Frequency of FIAS/** month*	**History of FS**
1	M	53	BHA (R > L)	B/B R > L	59	R CAH	6	OXC, LTG	No definite abnormality	Ia	9	7	No
2	F	12	LHA	L/L	58	L CAH	46	CBZ, CLB, LTG	MTS	Ia	7	2	No
3	M	3	LT pole resection LHA	None/L	51	L CAH	48	DPH, LEV, OXC, CLB	MTS	IV	3	3	No
4	F	16	LHA	L/L	65	L CAH	49	LTG, DPH, TPM, CLB	No definite abnormality	Ia	7	4	No
5	F	6	LHA	L/L	29	L CAH	23	PB	No definite abnormality	Ia	0.5	4	No
6	F	30	LHA	B/B (SEEG: B/L)	43	L SAH	13	TPM, LTG	MTS	II	2	6	No
7	M	44	BHA L>R	L/L	51	L SAH	7	CBZ, CLB, LEV	MTS	Ia	4.5	4.5	Yes
8	F	32	LHA	B/L	47	L CAH + ATL	15	OXC	MTS	Ia	9	1	Yes
9	F	before 7	RT resection RH signal abnormality	NA	26	R ATL	20	CBZ, CLB	Low grade astrocytoma FCD III	Ia	4	4	No
10	M	5	RHA	R	55	R ATL	50	OXC, LEV, CLB	MTS	Ia	6	9	No
11	F	8	L HMF LF encephalocele	L/L (SEEG: L/L)	39	L CAH	31	LEV, LCM	Gliosis	Ia	6	6	No
12	F	40	LHA	L	42	L SAH	2	CBZ, CLB	Gliosis	II	2	16	No
13	F	13	RHA	R	29	R SAH	16	LTG, CLB	MTS	Ia	9	2	No
14	M	13	BHA L > R + LFT encephalomalacia	L	67	L CAH	54	CBZ, LEV	Gliosis	Ia	7	1	No
15	F	4	RHA	R	36	R ATL	32	CBZ	MTS	Ia	6	2.5	No
16	F	16	RHA	R	66	R CAH	50	CBZ, CLB	MTS	Ia	1.5	2.5	No

*M, male; F, female; MRI, magnetic resonance imaging; B, bilateral; L, left; R, right; H, hippocampal; HA, hippocampal atrophy; HMF, hippocampal malformation/malrotation; T, temporal; F, frontal; EEG, electroencephalography; SEEG, stereo-EEG; NA, not available; CAH, cortico-amygdalo-hippocampectomy; SAH, selective amygdalo-hippocampectomy; ATL, anterior temporal lobe resection (including amygdala and hippocampus); CBZ, carbamazepine; CLB, clobazam; DPH, phenytoin; LEV, levetiracetam; LTG, lamotrigine; OXC, oxcarbazepine; LCM, lacosamide; PB, phenobarbital; TPM, topiramate; MTS, mesial temporal sclerosis; FCD III, focal cortical dysplasia type III*;

^*^Focal impaired awareness seizures (FIAS) per month in the year prior to surgery; FS, febrile seizures.

A total of 7 non-epilepsy control hippocampal samples from necropsy (aged 18–88 at time of death; 2 female) were procured from the Douglas-Bell Canada Brain Bank (Table 2). Characteristics of 3 of the controls in this study have been previously presented in a table by Vigneault et al. (27). Prior to the autoradiography experiment, flash frozen tissue blocks were cut into serial 20μm-thick sections, thaw-mounted on microscope slides, and stored at −80°C.

**Table 2:** Non-epilepsy controls from necropsy.

**ID**	**Gender**	**Age at death (years)**	**Post-mortem delay (hours)**	**Cause of death**
1	F	66	57.6	Traumatic injury from motor vehicle accident*
2	M	18	2	Natural death cardiovascular*
3	M	88	8	Ruptured abdominal aortic aneurysm*
4	M	59	17.67	Gastric neoplasm*
5	M	31	9.2	Natural death, cause unknown*
6	F	51	26.25	Breast cancer*

*M, male; F, female*;

^*^As recorded by the coroner.

### [^3^H]ABP688 Autoradiography

On the day of the binding assay, the frozen tissue sections were thawed to room temperature and pre-incubated in an Na HEPES buffer solution (30 mM Na HEPES, 110 mM NaCl, 5 mM KCl, 2.5 mM CaCl_2_, 1.2 mM MgCl_2_) with a pH of 7.4 (adjusted by NaOH) for 20 mins. The sections were left to air-dry then incubated for 60 mins in the same buffer solution containing one of 6 different concentrations (0.25–8 nM) of [^3^H]ABP688 (American Radiolabelled Chemicals; ARC). Non-specific binding was assessed *via* simultaneous incubation with 2-Methyl-6-(phenylethynyl)pyridine (MPEP), at a concentration of 10 μM, in 3 sections per specimen. Following incubation with [^3^H]ABP688, the sections were washed 3 times successively (5 mins/each) in ice-cold incubation buffer, then dipped in ice-cold distilled water for 30 s. The sections were air dried, then placed in a desiccator with paraformaldehyde overnight (16 h) for mild fixation. Slides were then exposed on tritium-sensitive phosphor imaging plates (Fujifilm) for 1 week alongside industrial tritium standards (ARC). The plates were imaged using an Amersham Typhoon biomolecular imager (spatial resolution 25 μm).

### Image Analysis

The average gray value per square pixel was measured for each region of interest (ROI) using ImageJ software (Fiji, https://imagej.net/software/fiji/, RRID:SCR_002285). Not all hippocampal subfields were clearly visible in all mTLE hippocampal surgical specimens; the ROI included the dentate gyrus, cornu ammonis (CA) areas, and subiculum, when identifiable. Radioactivity concentrations in the tissue (nCi/mg) were determined using a calibration curve constructed from the tritium standards, with values corrected for tissue equivalency. Receptor concentrations were expressed in fmol/mg using the specific activity of the [^3^H]ABP688 sample (80 Ci/mmol). Total and non-specific binding data were input into a one-site saturation binding model (GraphPad Prism, RRID:SCR_002798) to determine specific binding. Saturation binding parameters B_max_ (maximum specific binding) and K_D_ (dissociation constant) were extrapolated from the saturation binding curve. The maximum specific binding observed typically corresponds to the density of receptors.

### Statistical Analysis

Statistical differences in B_max_ between mTLE and control specimens were determined using a two-tailed t-test. Due to a large difference in sample sizes between the groups, Welsh's correction was applied despite meeting the equality of variance assumption. K_D_ values were non-normally distributed, therefore, group differences were compared using a two-tailed Mann-Whitney U Test. A multiple linear regression model was applied to the B_max_ data, with group (mTLE or control), age, sex, and K_D_ as predictors, to assess B_max_ changes between the groups independent of age, sex, and K_D_. Correlations between B_max_ and the age at surgery, age at seizure onset, duration of epilepsy (years until surgery), and frequency of seizures (focal impaired awareness seizures per month in the year prior to surgery) of mTLE patients (Table 1) were assessed with a Pearson or Spearman correlation. Differences in B_max_ and K_D_ were also assessed between patients who achieved seizure freedom following surgery (*n* = 13) and patients who did not achieve seizure freedom (*n* = 3), and between patients with a history of febrile seizures (*n* = 2) and patients who did not have a history of febrile seizures (*n* = 14) using Mann-Whitney U Tests. Seizure freedom status was determined by Engel classification (Ia = seizure free; II–IV = non-seizure-free).

## Results

One control specimen (male, age 61yo) was excluded from all analyses due to abnormally high [^3^H]ABP688 binding in the 0.5 nM condition (approximately equal to the 2nM condition), constituting an outlier. There were no significant differences in sex distribution [$\chi_{\left(1\right)}^{2}$ = 2.264, *p* = 0.132] and age [t_(6.132)_ = 0.416, *p* = 0.692] between control and mTLE groups. Both Age [r_(4)_ = −0.5061, *p* = 0.3056] and PMD [r_(4)_ = −0.1533, *p* = 0.7718] were uncorrelated with B_max_ in the control group, supporting their use as a control for the mTLE surgical samples.

### [^3^H]ABP688 Binding Site Is Reduced in mTLE: Comparison of B_max_

**Figure 1:**
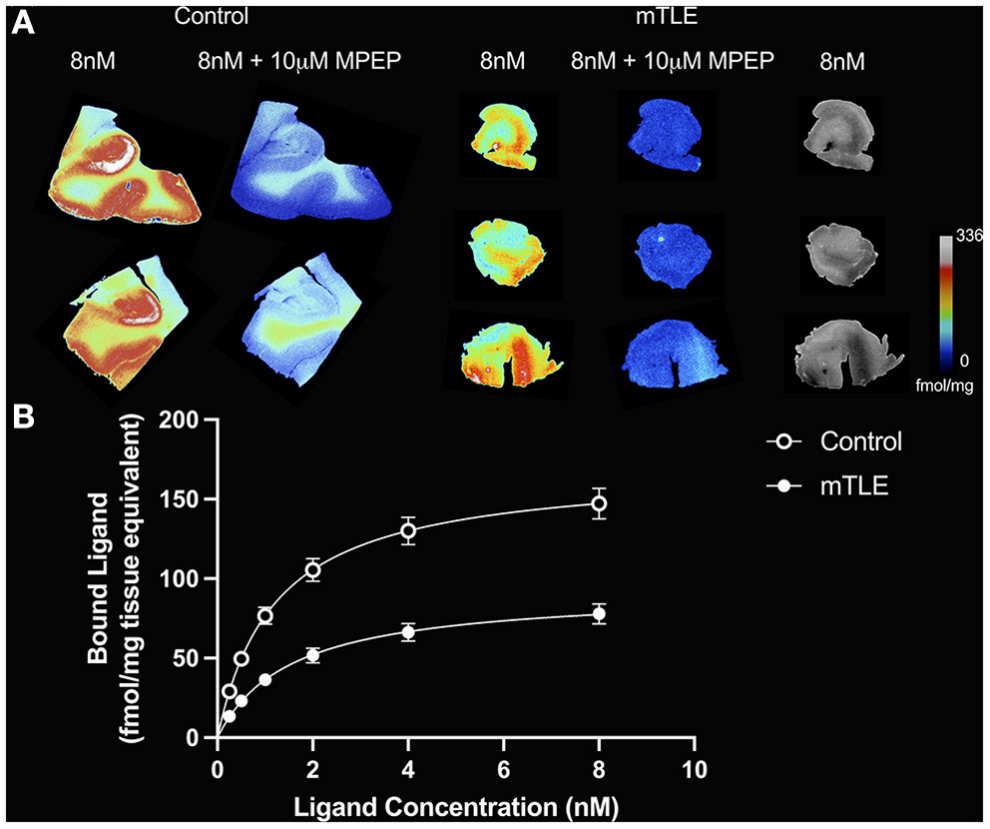
[^3^H]ABP688 binding in the hippocampus. **(A)** Saturation autoradiography with [^3^H]ABP688 in the hippocampus of two non-epilepsy necropsy controls and three mesial temporal lobe epilepsy (mTLE) patients. 10 μM of 2-Methyl-6-(phenylethynyl)pyridine (MPEP) was used to assess non-specific binding. Greyscale images with 8nM [^3^H]ABP688 demonstrate hippocampal structure in resected tissue from mTLE patients. **(B)** Autoradiographic saturation binding curves constructed from total and non-specific binding data. Displayed are the mean curves for specific binding of mGluR5 by [^3^H]ABP688 in hippocampal specimens from mTLE patients and non-epilepsy controls. Error bars represent SEM.

**Figure 2:**
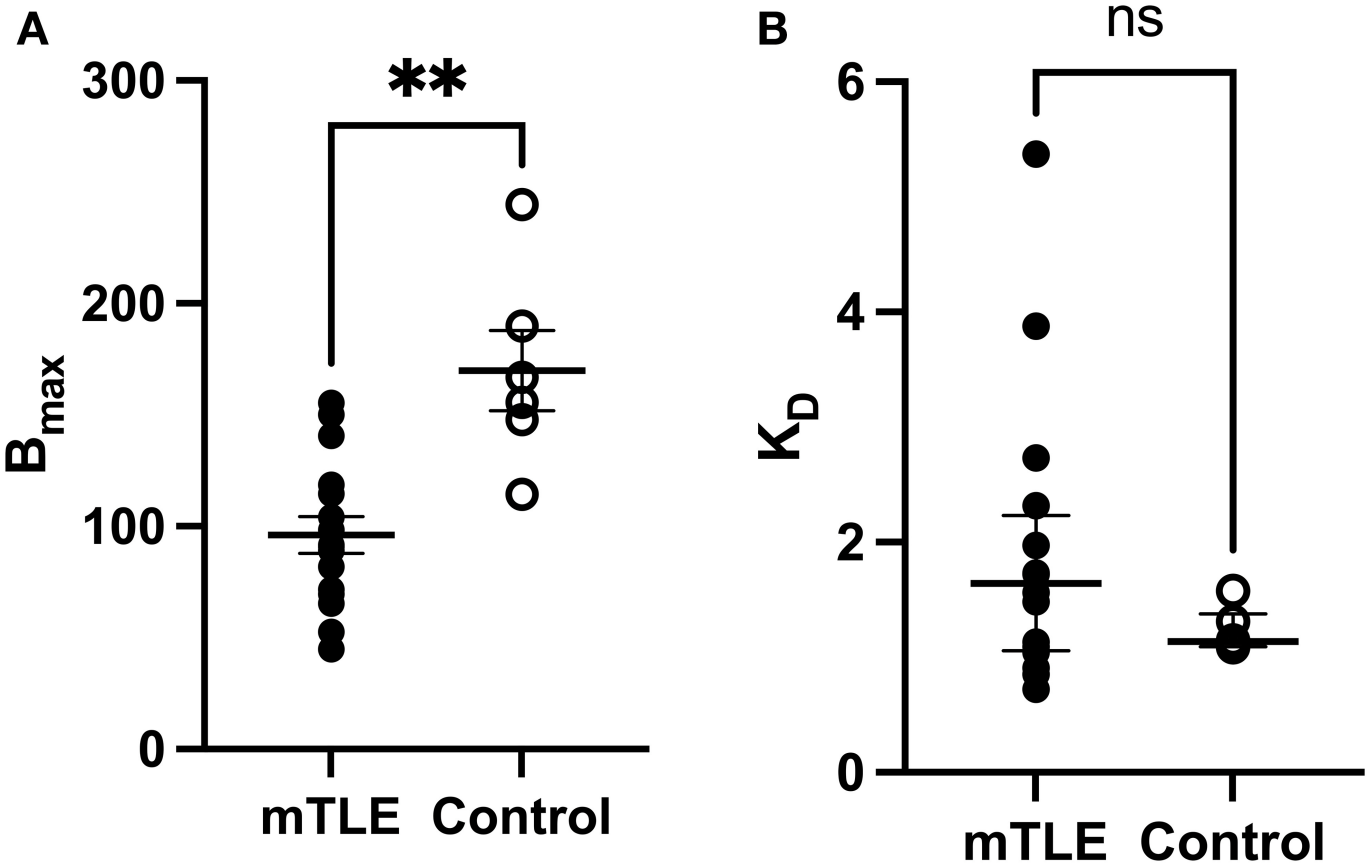
[^3^H]ABP688 B_max_ and K_D_ values in the hippocampus. Receptor densities (B_max_; in fmol/mg) and dissociation constants (K_D_) in hippocampal specimens from mesial temporal lobe epilepsy (mTLE) patients and healthy control individuals. **(A)** B_max_ values were reduced in mTLE patients compared to controls. Values are represented as mean ± std. error. **(B)** Dissociation constants were not significantly altered in mTLE patients compared to controls. Values are represented as median with interquartile range. **p < 0.01; ns, indicates a non-significant comparison.

A two-tailed unpaired t-test with Welsh's correction revealed significantly lower mean receptor density (B_max_) between mTLE patients and controls: t_(7.224)_ = 3.717; *p* = 0.007; n_mTLE_ = 16, n_control_ = 6. On average, B_max_ was 96 ± 33 fmol/mg tissue equivalent (mean ± SD) for mTLE patients and 170 ± 44 fmol/mg tissue equivalent for controls (Figures 1, 2A).

### [^3^H]ABP688 Affinity for MGLUR5 Does Not Change in mTLE: Comparison of K_D_

A two-tailed Mann-Whitney U Test revealed that the median K_D_ value in the mTLE group did not significantly differ from the median K_D_ value in the control group: U_(6, 16)_ = 33; *p* = 0.294. The median K_D_ was 1.640 (IQR = 2.059–1.081) for mTLE patients and 1.137 for controls (IQR = 1.271–1.102) (Figure 2B).

### Multiple Linear Regression Analysis of B_max_

Multiple linear regression analysis of B_max_ with group (mTLE vs. control), age, sex, and K_D_ as predictors yielded a significant model that explained 54% of the variance of B_max_: adjusted R^2^ = 0.5428; F_(4, 17)_ = 7.232, *p* = 0.001362. Further analysis of the model revealed that group was the only significant predictor of B_max_: β(mTLE) = −72.7288, *p* = 0.000578, 95% CI [−109.1007, −36.3569].

### Correlations of B_max_ With mTLE Patient Characteristics

**Figure 3:**
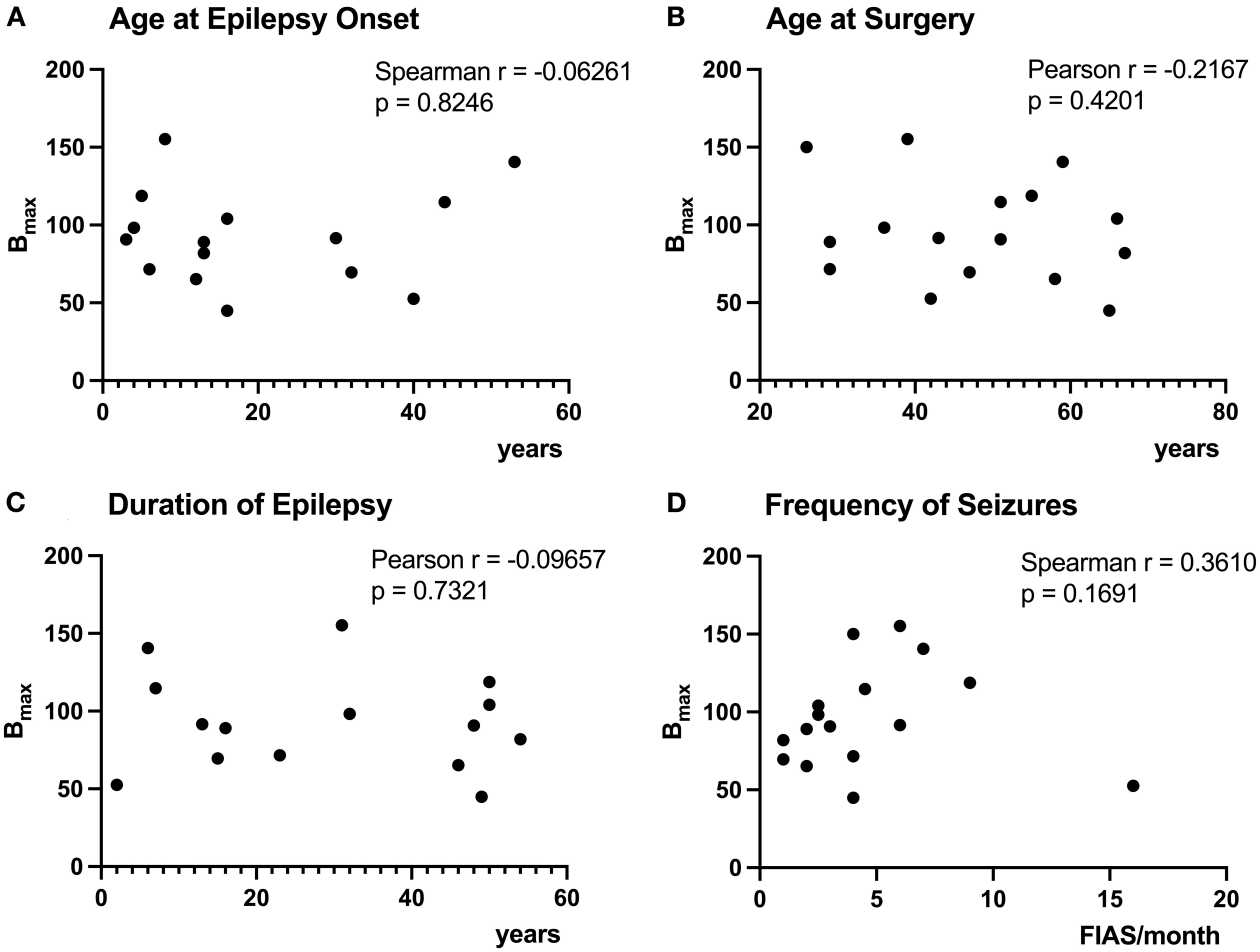
Correlation of [^3^H]ABP688 B_max_ and clinical demographics. Correlations between receptor densities (B_max_; in fmol/mg) and age at epilepsy onset **(A)**, age at surgery **(B)**, duration of epilepsy **(C)**, and frequency of seizures (focal impaired awareness seizures, FIAS, per month in the year prior to surgery) **(D)** are presented. The age at epilepsy onset was uncertain for P10, therefore, the patient was excluded from the analyses of B_max_ with age at epilepsy onset and epilepsy duration **(A,C)**. All correlations were non-significant with *p* > 0.05.

The age at epilepsy onset for patient 10 was before 7yo, but exact age was uncertain, therefore, we have excluded the patient from the analyses of B_max_ with age at onset and epilepsy duration. Correlations between B_max_ and duration of epilepsy, age at seizure onset, age at surgery, and frequency of seizures were all non-significant (Figures 3A–D).

**Figure 4:**
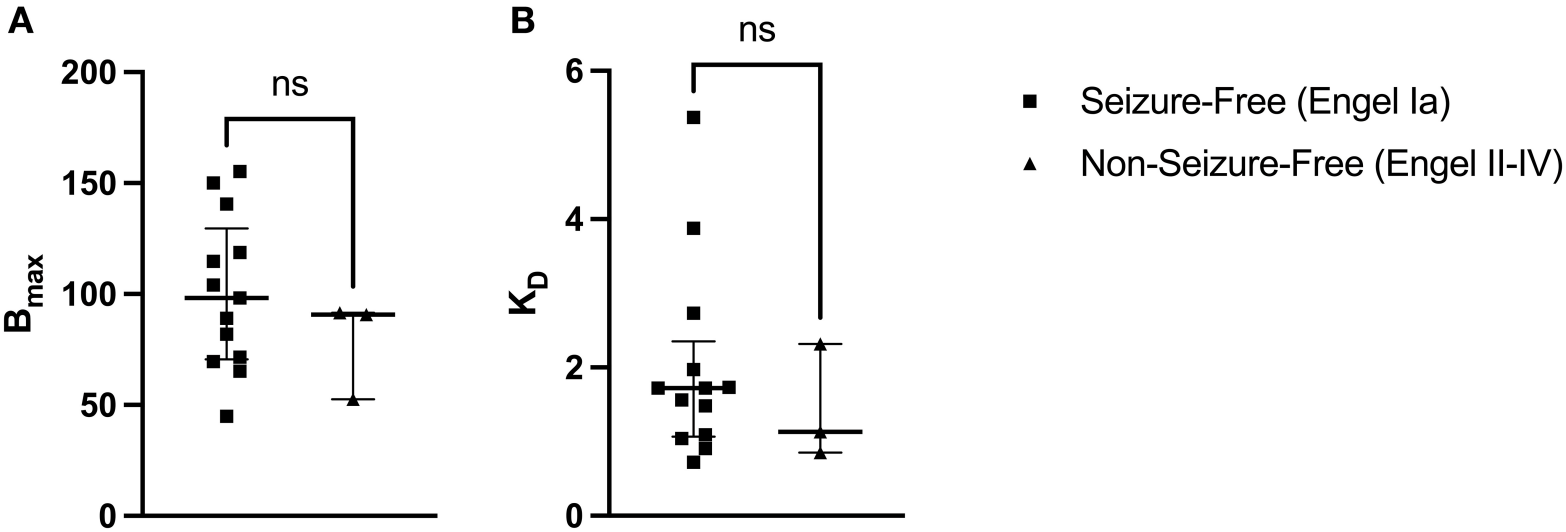
[^3^H]ABP688 B_max_ and K_D_ in seizure-free (Engel Ia) and non-seizure-free (Engel II–IV) patients. Receptor densities (B_max_; in fmol/mg) and dissociation constants (K_D_) in hippocampal specimens from mesial temporal lobe epilepsy (mTLE) patients who achieved seizure freedom following surgery and patients who did not achieve seizure freedom. **(A)** B_max_ values were not significantly different between seizure-free and non-seizure-free groups. **(B)** K_D_ values were not significantly different between seizure-free and non-seizure-free groups. Values are represented as median with interquartile range; ns, indicates a non-significant comparison.

A two-tailed Mann-Whitney U Test revealed that the median B_max_ value in the seizure-free group (Engel Ia) was not significantly different than the median B_max_ value in the non-seizure-free group (Engel II–IV): U_(13, 3)_ = 13, *p* = 0.4393 (Figure 4A). The median B_max_ was 98.290 fmol/mg (IQR = 118.700–71.570) for seizure-free patients and 90.820 fmol/mg (IQR = 91.165–71.705) for non-seizure-free patients. Similarly, the median K_D_ value in the seizure-free group was not significantly different than the median K_D_ value in the non-seizure-free group: U_(13, 3)_ = 15, *p* = 0.6107 (Figure 4B). The median K_D_ was 1.720 (IQR = 1.974–1.094) for seizure-free patients and 1.134 (IQR = 1.725–0.994) for non-seizure-free patients.

**Figure 5:**
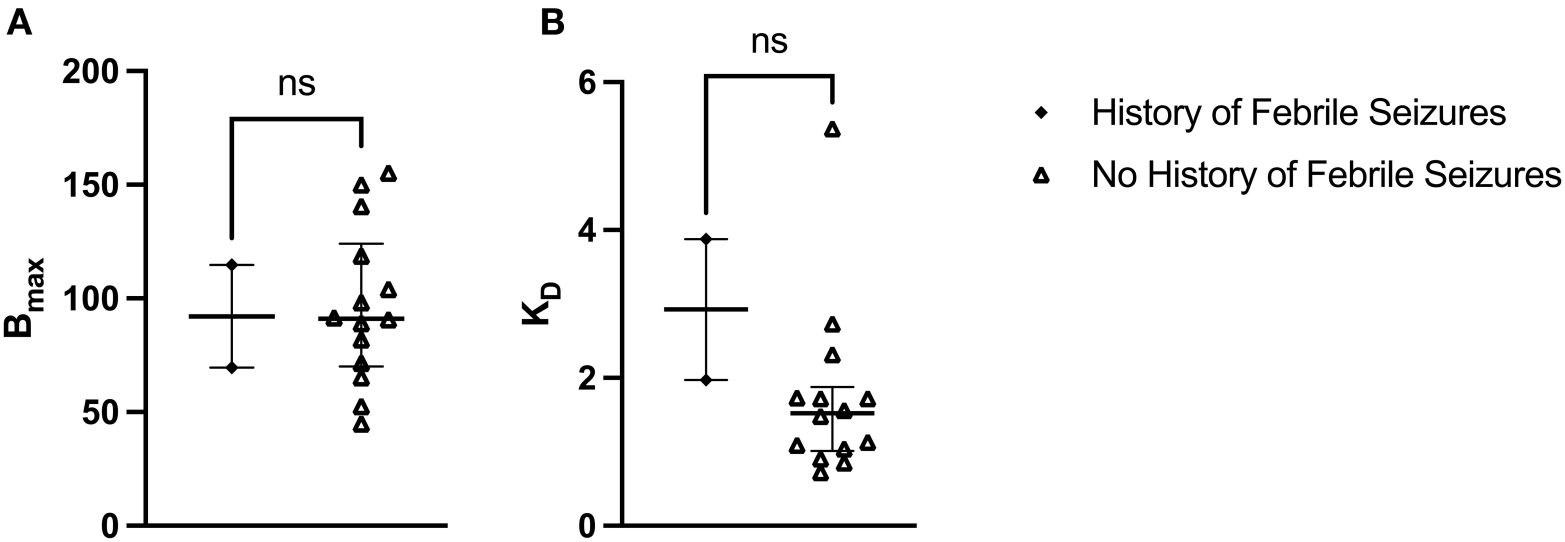
[^3^H]ABP688 B_max_ and K_D_ in patients with and without a history of febrile seizures. Receptor densities (B_max_; in fmol/mg) and dissociation constants (K_D_) in hippocampal specimens from mesial temporal lobe epilepsy (mTLE) patients with or without a history of febrile seizures. **(A)** B_max_ values were not significantly different between patients with a history of febrile seizures and patients with no history of febrile seizures. **(B)** K_D_ values were not significantly different between patients with a history of febrile seizures and patients with no history of febrile seizures. Values are represented as median with interquartile range; ns, indicates a non-significant comparison.

The median B_max_ value for patients with a history of febrile seizures was not significantly different from the median B_max_ value for patients with no history of febrile seizures: U_(2, 14)_ = 15, *p* = 0.933 (Figure 5A). The median B_max_ was 92.115 fmol/mg (IQR = 103.407–80.823) for patients with a history of febrile seizures and 91.165 fmol/mg (IQR = 115.050–74.140) for patients with no history of febrile seizures. Similarly, the median K_D_ value for patients with a history of febrile seizures was not significantly different from the median K_D_ value for patients with no history of febrile seizures: U_(2, 14)_ = 4, *p* = 0.150 (Figure 5B). The median K_D_ was 2.926 (IQR = 3.402–2.450) for patients with a history of febrile seizures and 1.522 (IQR = 1.730–1.056) for patients with no history of febrile seizures.

## Discussion

In this study we deployed gold standard techniques to quantify mGluR5 density in hippocampal tissue using quantitative autoradiography. We found reduced density in mTLE hippocampi as compared to controls. mTLE did not impose changes on [^3^H]ABP688 affinity for mGLUR5. [^3^H]ABP688 density observed in mTLE hippocampi in this *ex vivo* study corroborates previous observations describing reduced [^11^C]ABP688 BP *in vivo* in pilocarpine-treated rats and in the epileptogenic hippocampi of mTLE patients using PET (18, 19). The present results corroborate *in vivo* [^11^C]ABP688 PET findings of reduced availability of mGluR5 in the epileptogenic hippocampus in mTLE. In the context of previous observations from independent studies using mGluR5 IHC, membrane-expressed receptor pools with and without conformational changes, as well as internalized receptors, may contribute to the observation of increased immunoreactivity in the tissue.

The magnitude of the B_max_ reduction, of over 40% as compared to control specimens, is well aligned with *in vivo* observations in mTLE patients using [^11^C]ABP688 PET (18). In addition, the lack of a significant difference in K_D_ values suggests the binding affinity of [^3^H]ABP688 for mGluR5 is similar between mTLE and control specimens. Lam and colleagues suggested that excess glutamate binding to mGluR5 may reduce [^11^C]ABP688's affinity for its allosteric binding site, contributing to reduced BP *in vivo* (18). Although our [^3^H]ABP688 autoradiography protocol includes a pre-incubation step that removes endogenous ligands from the tissue, brain cells have been flash-frozen immediately after surgery, and mGluR5 conformation therefore remains static as a snapshot at time of resection. However, the preservation of K_D_ indicates that, at the time of resection, the pool of receptors to which [^3^H]ABP688 binds in the tissue had not undergone any glutamate-induced conformational changes affecting [^3^H]ABP688' affinity for mGluR5's allosteric site.

Although reduced [^3^H]ABP688 binding could be at first interpreted as reduced mGluR5 expression in mTLE hippocampi, previous mGluR5 IHC studies have shown increased immunoreactivity which indicates increased mGluR5 expression instead (13, 14). Even though neither necropsy controls nor patients' specimens had mGluR5 IHC to support that, this apparent discrepancy can be easily explained by the techniques applied. First, the fixation and permeabilization steps of IHC studies permit detection of internalized as well as membrane-bound receptors (18, 28). The antibodies used in the studies by Kandratavicius, Notenboom and their respective colleagues that demonstrated mGluR5 upregulation targeted the cytoplasmic domain of the receptor, whereas ABP688 targets the transmembrane domain (13, 14, 23). If a conformational change affecting the availability of the transmembrane allosteric binding site, but not the C-terminal antibody binding site, occurred, then it would be possible to observe decreased [^11^C] and [^3^H]ABP688 binding with PET and autoradiography despite upregulation of mGluR5. As such, [^3^H]ABP688 binding could be interpreted as a marker of a specific pool of mGLUR5 receptors.

Notably, intracellular mGluR5 has recently emerged as an important modulator of synaptic plasticity (29–31). For example, local dendritic calcium increases and protein-synthesis-dependent LTD, but not LTP, were shown to be mediated by intracellular mGluR5 (32). However, further research is needed to discern whether intracellular mGluR5 expression is altered in mTLE.

As previously discussed, [^3^H]ABP688 requires the allosteric site in the TM domain to be accessible for binding, and membrane expressed mGluR5 might undergo conformational changes that renders the binding site unavailable for the ligand. In addition, due to chemical and physical differences between the cytoplasmic and membrane milieux, it is unknown whether [^3^H]ABP688 can detect mGluR5 that is dissociated from the cell membrane, thus remaining unclear if it can bind to internalized mGluR5 (33).

The decreases in [^11^C] and [^3^H]ABP688 binding observed *in vivo* and *ex vivo* seem instead to reflect a scenario in which mGluR5 allosteric binding sites are not available for ligand binding. Access into the binding pocket within the allosteric site for mGluR5 negative allosteric modulators has been shown to be narrow and restricted (34). Additionally, an inverse relationship between extracellular glutamate levels and [^11^C]ABP688 binding has been observed (35). Subsequently, glutamate-induced conformational changes that lead to occlusion of this binding site may explain reduced availability in a brain region that is under recurrent hyperexcitability, such as the epileptic focus.

Furthermore, mGluR5 is expressed as a constitutive dimer, and the dimer structure of mGluRs has been shown to influence the activity of allosteric modulators (36). Indeed, agonist binding has been shown to lead to a rearrangement at the 7TM dimer interface of mGluRs (37). Therefore, it is conceivable that an excess glutamate-induced change to mGluR5 dimerization state could elicit changes in the availability of the allosteric binding site. Furthermore, the monomer-dimer ratio of mGluRs has been shown to be affected in other neurological disorders and could well occur in epilepsy as well (38, 39). If the dimerization of mGluR5 were essential for ABP688 binding, then the observed decreases could also reflect a shift in the ratio of monomeric and dimeric mGluR5 in mTLE.

The interpretation of these results should take into considerations a few methodological limitations. The present study did not evaluate region-specific differences in mGluR5 density and binding affinity due to an incomplete view of hippocampal subregions in mTLE specimens. Future studies could investigate regional differences to identify possible hippocampal subfield-specific mechanisms underlying mTLE. Additionally, emerging evidence that mGluR5 has a variety of functions within the cell also requires further research into how intracellular pathways may be involved in epileptogenesis or neuroprotection. Most of the current literature on mGluR5 has focused on the function of the receptor when it is associated with the cell membrane, so exploring how this protein behaves when located intracellularly could offer new insight into this topic.

This study has demonstrated that [^3^H]ABP688 binding, but not binding affinity, is altered in the hippocampus of mTLE patients. We propose that these reductions signify the internalization of mGluR5 or a loss of allosteric binding site availability related to conformational alterations of mGluR5.

## Data Availability Statement

The raw data supporting the conclusions of this article will be made available by the authors, without undue reservation.

## Ethics Statement

The studies involving human participants were reviewed and approved by MNI/McGill MUHC REB. The patients/participants provided their written informed consent to participate in this study.

## Author Contributions

Study design and data analysis: MZ, LM, PR-N, and EK. Data acquisition: MZ and AA. Manuscript preparation: MZ and EK. Manuscript revision and approval: All authors. All authors contributed to the article and approved the submitted version.

## Funding

This study was supported by operating funds from the Savoy Foundation (www.savoy-foundation.ca; Pilot project grant to EK, PR-N, and MSc studentship to MZ).

## Conflict of Interest

The authors declare that the research was conducted in the absence of any commercial or financial relationships that could be construed as a potential conflict of interest.

## Publisher's Note

All claims expressed in this article are solely those of the authors and do not necessarily represent those of their affiliated organizations, or those of the publisher, the editors and the reviewers. Any product that may be evaluated in this article, or claim that may be made by its manufacturer, is not guaranteed or endorsed by the publisher.
